# Lawyers and Doctors

**Published:** 1910-02

**Authors:** 


### Lawyers and Doctors

THE New York State Bar Association held its thirty-third annual meeting at Rochester, January 20, 21, 1910. With all due respect to the eminent legal gentlemen who made up the committee that furnished the report on the committment and discharge of the criminal insane, the JOURNAL cannot well allow such an uncalled for and unjust attack upon the medical profession to pass unnoticed. The sins of a few are not the sins of all, either in the legal profession or in that of medicine. In a book that generally commands respect, and with which we are sure the Rochester committee is entirely familiar, there is a reference made to a certain mote that found lodgement in a gentleman's eye, which was evidently not considered by the assembled lawyers who met in our sister city.

The medical profession as a profession, does not in any respect occupy a lower level in our social fabric than that of the law. The great mass of both callings is made up of earnest, sincere and honorable citizens, mindful alike of what constitutes right, decency and justice. Each is striving to do his part, and each willing and ready to aid others in bringing about a condition of affairs in his community, that will meet the approval of respectable people everywhere. For this he does not ask, nor does he deserve any special credit. It is a part of his duty and an evidence of good citizenship.

That there are bad and unscrupulous men in both callings, and in all callings for that matter, goes without saying. Of all the dozen or more socalled medical experts summoned to give evidence on either side in the case of Thaw—cited in the report—there was but one physician who did not acquit himself, let us say with a reasonable amount of credit and that one has never had a high standing in the profession of medicine, not being recognised as an authority on any scientific subject. The medical profession has no apology to make for him,—does not in any way shield him,—and believes he deserves all the criticism and ridicule he received. The brain-storm created in his case, by the sight of the plethoric Thaw pocket book, was evidently another instance of the presence of the serpent in the garden of Eden.

It is evident that the methods now existing in admitting medical expert testimony in civil and criminal trials, do not effectively subserve the ends of justice, and are alike unsatisfactory to the judges, the bar, and the medical men. Would it not clear the atmosphere and be the better way for the two great professions of the state of New York, to unite in a common cause and exert their combined influence with our present law-making power, to the end that proper laws be enacted which will bring about a better understanding, and a more satisfactory condition than now exists?

---

THE Metropolitan Life Insurance Company of New York has compiled, printed, and distributed a brochure containing a list of the tuberculosis sanatoria, hospitals, dispensaries, classes, and associations in Canada, New Jersey, New York, and Pennsylvania. It is accompanied by a pamphlet entitled "A war upon consumption," which to a certain degree explains the nature of the disease, its extent, growth and spread; its cure and prevention, including friendly advice to persons having diseases of the lungs. The latter is illustrated by a number of cuts that bear upon the methods of spreading tuberculosis. Both pamphlets are intended for distribution among the policy holders of the company that issues them. They are instructive, however, to the laity in general, and can be obtained by addressing Dr. Lee K. Frankel, Manager Industrial Department, Metropolitan Life Insurance Company, New York.

---

IN February, 1909, Surgeon C. P. Wertenbaker of the Public Health and Marine Hospital Service, in giving an illustrated lecture on tuberculosis before the Negro Farmers' Conference at Savannah, Ga., suggested the organisation of a State Antituberculosis League for Negroes. The idea was well received and a league was organised. The proposed plan of organisation contemplated a league in each state, with a branch in every colored church. This plan, which has been followed, is given in detail in the Public Health Reports of May 28, 1909. The movement was indorsed by the last conference of state and territorial boards of health.

Up to August 6, leagues had been formed in the following states: Georgia, Louisiana, Mississippi, North Carolina and Virginia. "A Working Plan" for these leagues has been published in the Public Health Reports of September 3, 1909, giving in detail the method of organisation of state leagues and of the local branch leagues.

The "Proposed Plan of Organisation," and the "Working Plan" have been reprinted, and limited editions are available for distribution to those interested in the work. Requests for copies should be addressed to the Surgeon-General, Public Health and Marine-Hospital Service, Washington, D. C.

---

In the Public Health Reports for August 27, 1909, appears an article on "Plague among Ground Squirrels in Contra Costa County, California," written by W. C. Rucker, passed assistant surgeon. In 1894 plague began to spread from central Asia. Since then it has been carried to practically all parts of the world, including the Pacific Coast of the United States, where the disease has appeared in man, in rats, and in ground squirrels. The infection in ground squirrels has so far appeared in Contra Costa and Alameda Counties, California, chiefly in the former, where, up to September 10, 1909, 220 plague infected squirrels had been found. The Public Health and Marine-Hospital Service is attempting to destroy all the ground squirrels in the involved area, or at least to so reduce them in number that the plague infection among them will die out of its own accord. This article gives a detailed account of plague infection among the ground squirrels in Contra Costa County, and the relation of squirrel plague to plague in man. It also describes the means employed for the destruction of the squirrels, and gives a serial list of infected squirrels with the location where found.

The article has been reprinted, and a limited edition is available for distribution to those interested. Requests for copies should be made to the Surgeon-General, Public Health and Marine-Hospital Service, Washington, D. C.

---

It may be possible most persons have overlooked the fact that a revolution occurred in the Argentine Republic a century ago. Be that as it may that country proposes to commemmorate the first centenary of the revolution in the city of Buenos Aires, May 25, 1910, at which time an International American Congress of medicine and hygiene will be held. An exhibition of hygiene also will take place. A pamphlet descriptive of the scheme has been distributed, and which contains many illustrations of interest such as the Plaza 25 de Mayo, or Chief Public Square; the Parliament Building, the Medical Faculty Building, several theatres, and landscapes showing the prominent features of the city of Buenos Aires. Elsewhere will be found an announcement of the Congress under its appropriate heading, giving the officers appointed for this country. Plans of the city of Buenos Aires and of the grounds and buildings of the congress are also given, as well as much other material of interest.

